# Constitutive Activation of the Midgut Response to *Bacillus thuringiensis* in Bt-Resistant *Spodoptera exigua*

**DOI:** 10.1371/journal.pone.0012795

**Published:** 2010-09-17

**Authors:** Patricia Hernández-Martínez, Gloria Navarro-Cerrillo, Silvia Caccia, Ruud A. de Maagd, William J. Moar, Juan Ferré, Baltasar Escriche, Salvador Herrero

**Affiliations:** 1 Department of Genetics, Universitat de València, Burjassot, Spain; 2 Plant Research International B.V., Wageningen University and Research Centre, Wageningen, The Netherlands; 3 Department of Entomology and Plant Pathology, Auburn University, Auburn, Alabama, United States of America; Instituto Butantan, Brazil

## Abstract

*Bacillus thuringiensis* is the most effective microbial control agent for controlling numerous species from different insect orders. The main threat for the long term use of *B. thuringiensis* in pest control is the ability of insects to develop resistance. Thus, the identification of insect genes involved in conferring resistance is of paramount importance. A colony of *Spodoptera exigua* (Lepidoptera: Noctuidae) was selected for 15 years in the laboratory for resistance to Xentari™, a *B. thuringiensis-*based insecticide, reaching a final resistance level of greater than 1,000-fold. Around 600 midgut ESTs were analyzed by DNA-macroarray in order to find differences in midgut gene expression between susceptible and resistant insects. Among the differentially expressed genes, *repat* and *arylphorin* were identified and their increased expression was correlated with *B. thuringiensis* resistance. We also found overlap among genes that were constitutively over-expressed in resistant insects with genes that were up-regulated in susceptible insects after exposure to Xentari™, suggesting a permanent activation of the response to Xentari™ in resistant insects. Increased aminopeptidase activity in the lumen of resistant insects in the absence of exposure to Xentari™ corroborated the hypothesis of permanent activation of response genes. Increase in midgut proliferation has been proposed as a mechanism of response to pathogens in the adult from several insect species. Analysis of *S. exigua* larvae revealed that midgut proliferation was neither increased in resistant insects nor induced by exposure of susceptible larvae to Xentari™, suggesting that mechanisms other than midgut proliferation are involved in the response to *B. thuringiensis* by *S. exigua* larvae.

## Introduction

*Bacillus thuringiensis*-based biopesticides are employed for the control of numerous species from Lepidoptera, Diptera, and Coleoptera [1]. The pathological effect of *B. thuringiensis* is mediated by the effect of crystal (Cry) proteins produced during sporulation, and accumulating as crystalline inclusions. Cry proteins are pore forming toxins that, after their solubilization and activation in the insect midgut, bind to specific receptors in the microvilli of midgut epithelial cells. The toxins are highly specific to some insects and are innocuous to humans, other vertebrates and plants [2]. These characteristics have made them suitable for their use as the active agents in insect-resistant crops [3].

The ability of insects to develop resistance to *B. thuringiensis-*based formulated products and/or Cry proteins has been reported for many insect species, mostly in the order Lepidoptera [4]; [5]. To date, resistance to *B. thuringiensis-*based formulated products has only evolved in populations of *Plodia interpunctella* [6], *Plutella xylostella* [4] and *Trichoplusia ni* [7], in stored grain and under field or greenhouse conditions, respectively. However, artificial selection using *B*. *thuringiensis* formulations or Cry proteins under laboratory conditions has generated *B. thuringiensis-*resistant colonies in many more insect species [5]. The most accepted and studied resistance mechanism is the reduction of Cry protein binding to the insect midgut. In some cases, this reduced binding is associated with mutations or altered expression of genes encoding cadherin, aminopeptidase N or alkaline phosphatase receptors [8]–[10]. These mechanisms that generally cause the highest levels of resistance have been usually found in insects selected for resistance to a single or few Cry proteins [4]; [11]. Other mechanisms of resistance in Lepidoptera have been associated with an alteration in proteolytic processing of Cry protoxins [12], sequestering of Cry proteins by midgut esterases [13] and lipophorin [14] and an increased recovery of midgut cells after Cry1Ac intoxication [15]. In *Caenorhabditis elegans* a defect in the synthesis of glycolipids that act as Cry5B receptors has been reported in Cry5B-resistant mutants [16]; [17].

Changes in the host's gene expression in response to bacterial or pore-forming toxins have been extensively monitored to identify genes and pathways involved in reducing the effect of the causal agent [18]; [19]. Most of these studies used whole organisms and only recently have these studies focused on the primary tissue associated with the penetration of bacteria into the insect: the gut. Moreover, these studies were performed in model invertebrates, such as *Drosophila melanogaster* [20] or *C. elegans* [21], with few studies carried out on Lepidopteran pests [22]; [23]. Recent studies show that changing midgut proliferation is an important mechanism to overcome or attenuate the effect of bacteria ingestion in *D. melanogaster* [20]; [24]; [25] and *Anopheles stephensi* [26] adults.

Resistance to *B. thuringiensis* can be multigenic in many cases, and even in those cases that seem to fit a monogenic model, resistance is rarely completely recessive [4]; [5], strongly suggesting that resistant phenotypes contain major and minor genes contributing to overall resistance. This fact is particularly relevant where virulence factors such as the bacterial spore play a vital role in the overall toxicity of *B. thuringiensis-*based insecticides [2] and becoming resistant to it may require from the contribution of more than one gene. In the present study we were interested in identifying altered gene expression correlated with resistance to a *B. thuringiensis-*based formulated product. For this purpose a DNA-macroarray was prepared with *Spodoptera exigua* midgut ESTs (expressed sequence tags) obtained from a suppression subtractive hybridization (SSH) library enriched for genes differentially expressed after feeding with *B. thuringiensis* Cry1Ca toxin [9]. We used the macroarray to compare midgut gene expression between a colony of *S. exigua* susceptible and a colony highly resistant to the *B. thuringiensis-*based formulated product, Xentari™ that contains Cry1Ca as the primary *S. exigua*-active Cry protein (Valent Bioscience Co., Libertyville). Results show strong over-expression of many genes that were also found up regulated in susceptible insects after exposure to sublethal concentrations of Xentari™. A strong correlation was also found between gene expression and the resistant phenotype.

## Results

### Continuous exposure to Xentari™ selects for resistance to *B. thuringiensis*

A colony of *S. exigua* was reared for 15 years on artificial diet containing 10 mg Xentari™/gram diet. Susceptibility of the selected colony (Xen-R) to Xentari™ was determined and compared with a susceptible colony (FRA) (Table 1). When measured as neonate mortality, Xen-R was more than 1,000-fold resistant to Xentari™ compared to FRA. Resistance in Xen-R also was detected in later instars when larvae are typically less susceptible to *B. thuringiensis* formulates (personal observation). Xen-R was 53-fold and 138-fold more resistant than FRA when growth inhibition GI_50_ and GI_95_ values were measured on fourth-instar larvae in 1-day bioassays, respectively. Reversion of resistance was also evaluated after 8 generations of rearing Xen-R in the absence of Xentari™ (Xen-RU). GI_50_values for Xen-RU revealed a significant reduction (Ancova test; *p-value* <0.05) in resistance levels when selection was discontinued (RR of 12 (GI_50_)- or 78-fold (GI_95_). These results indicate that resistance was not fixed, and that fitness costs are associated with Xen-R resistance.

**Table 1:** Toxicity of Xentari towards the different colonies from *S. exigua*.

Colony	LC_50_ (FL_95%_)^a^	RR_50_ b	GI_50_ (FL_95%_)^c^	RR_50_ b	GI_95_ (FL_95%_)^c^	RR_95_ b
FRA	1(0.4–2)	–	2 (1–6)	–	27.4 (11–139)	–
Xen-R	>1,000	>1,000	133 (46–383)	53	3767 (1069–42560)	138
Xen-RU	ND	ND	24 (5–62)	12	2118 (830–8851)	78

a LC_50_ (50% Lethal concentration) values were measured for neonate larvae. Concentrations are expressed as ng/cm^2^. FL_95%_ (95% Fiducial limit).

b The RR (resistance ratio) is obtained by dividing the LC_50_ or the GI of the resistant colony by the LC_50_ or the GI of the susceptible (FRA) colony.

c GI_50_ (50% growth inhibition) and GI_95_ (95% growth inhibition) values were measured for 4^th^ instar larvae. Concentration are expressed as µg/cm^2^.

ND, not determined.

### Novel repat genes and arylphorin are highly expressed in the Xen-R insects

A DNA-macroarray was prepared by spotting DNA derived from an SSH library enriched in genes differentially expressed in Cry1Ca-resistant *S*. *exigua* midgut after feeding with Cry1Ca toxin. Expression levels of 570 ESTs were compared between fourth instar larvae from susceptible (FRA) and Xen-R resistant colonies (not-exposed to Xentari™). From the 570 ESTs included in the array, 91 were differentially expressed (*p-value* <0.05) between the colonies. Among the differentially expressed ESTs, 75 (82%) were over-expressed in Xen-R (expression ratio ranged from 14- to 2.5-fold) and 16 (18%) were under-expressed in Xen-R (expression ratio ranged from 8- to 2.2-fold) (Table S1).

Due to the different origins of both colonies, small expression differences might be attributed to natural variation. We therefore focused our study initially on genes exhibiting strong (>10-fold) expression differences (Table 2). Six ESTs met this criterion, all with higher expression levels in Xen-R compared to FRA. Four of the 6 EST's encoded proteins identical or related to proteins previously reported to be up-regulated after bacterial feeding (arylphorin) or intoxication with *B. thuringiensis* toxins (REPAT) [23]; [27]. The 6 ESTs included: 1 EST representing *repat4* (Sex_SSH_44), 2 ESTs with homology to new members of the *repat* family (Sex_SSH_437 and Sex_SSH_471), one EST with homology to a *translation elongation factor* (Sex_SSH_52), one EST to a *triacylglycerol lipase* (Sex_SSH_225), and one EST to an *arylphorin subunit* gene (Sex_SSH_279) (Table 2). An additional EST (Sex_SSH_38) with homology to members of the *repat* family, but below the established 10-fold threshold (8.5-fold increase), was selected for subsequent studies as well because it represented a novel gene from the *repat* family.

**Table 2:** Relative expression of some selected genes differentially expressed between Xen-R and FRA insects.

	GenBank EST accession n°	BLASTX sequence homology (new name)		Expression Ratio (SD)	
EST			E-value	DNA-array	qRT-PCR
Sex_SSH_437	HO001693	*repat2*-ABO64232 (*repat6*)	3e-11	13.6 (0.7) 1	6.8 (7.3)^1^
Sex_SSH_471	HO001704	*repat4*-ABO64234 (*repat7*)	3e-24	10.9 (0.7) 1	5.6 (6.1)^1^
Sex_SSH_44	HO001694	*repat4*		10.6 (0.7) 1	5.7 (7.3)^1^
Sex_SSH_52	HO001719	*EF2*-AAL83698	2e-13	10.5 (0.7) 1	0.68 (0.3)
Sex_SSH_225	HO001778	*triacylglycerol lipase*-O46559	8e-08	10.2 (0.7) 1	1.3 (1.6)
Sex_SSH_279	HO004497	*arylphorin subunit*-CAB55605	2e-41	10.2 (0.7) 1	7.1 (8.9)^1^
Sex_SSH_38	HO001678	*repat2*-ABO64232 (*repat5*)	1e-8	8.5 (0.7) 1	46 (54)^1^
Sex_SSH_23	HO001634	*repat1*	–	1.4 (0.7)	3.7 (4.2)
Sex_SSH_83	HO001758	*repat2*	–	2.5 (0.7) 1	3.3 (4.5)
Not included	–	*repat3*	–	–	1.4 (1.8)

1 Expression ratio between the susceptible (FRA) and the resistant (Xen-R) insects was statistically different from 1 (*p-value* <0.05).

**Figure 1:**
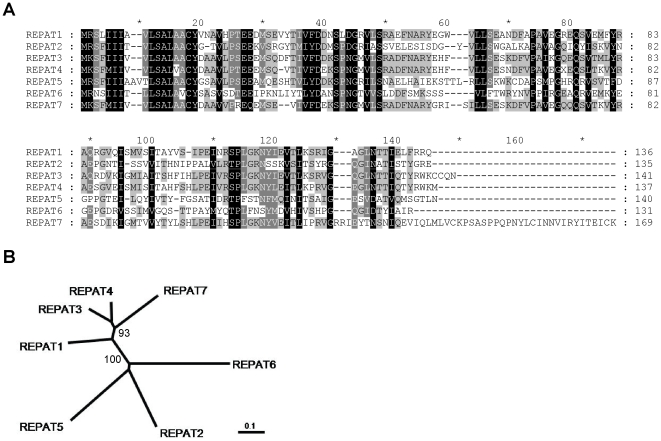
Sequence comparison of novel REPAT proteins. A) ClustalW alignment of the deduced amino acid sequences from novel REPAT proteins. (B) Phylogenetic tree derived from the ClustalW alignment by the neighbor-joining method. To clarify the figure, bootstrap values are shown only for the two main branches.

EST sequences with homology to *repat* genes were used to design specific (as well as nested) primers for amplification and cloning of overlapping 5′and 3′ cDNA fragments, which were sequenced and assembled into complete mRNA sequences. The sequenced cDNAs had total lengths of 564, 580, and 553 bp for *repat5* (GenBank FJ595234), *repat6* (GenBank FJ595235) and *repat7* (GenBank FJ595236), respectively. Proteins of 140, 131, and 169 amino acids were predicted from translation of their respective cDNA sequences (Fig. 1). As occurred with previously described REPAT proteins, analysis of new REPAT proteins predicted the presence of a secretory signal peptide in their N-terminal region. Screening of the predicted proteins for the presence of known domains against the Conserved Domain Database (CDD) (NCBI database) retrieved no significant homology. The predicted protein sequences and previously described REPAT protein sequences from *S. exigua*, were aligned using the Clustal W algorithm (Fig. 1A). Phylogenetic reconstruction by N-J trees (Fig. 1B) revealed the clustering of REPAT1, REPAT3, REPAT4 and REPAT7 proteins and a more divergent branch comprising the remaining *S. exigua* REPAT proteins.

Quantitative-PCR (qRT-PCR) was used to validate changes in expression levels obtained from DNA-macroarray experiments (Table 2). We included *repat1*, *repat2*, and *repat3* to provide a more accurate estimation of relevant differences in *repat* expression (Table 2). DNA-macroarray results were confirmed for all selected genes except for *translation elongation factor2* (*EF2*) and the *triacylglycerol lipase* homologs. Similar expression between FRA and Xen-R was found for *repat1*, *repat2*, in agreement with macroarray values (Table 2). For the *repat3* gene that was not included in the macroarray, qRT-PCR did not reveal any differences between both colonies.

### Midgut response to Xentari™ is activated in Xen-R without exposure to Xentari™

**Figure 2:**
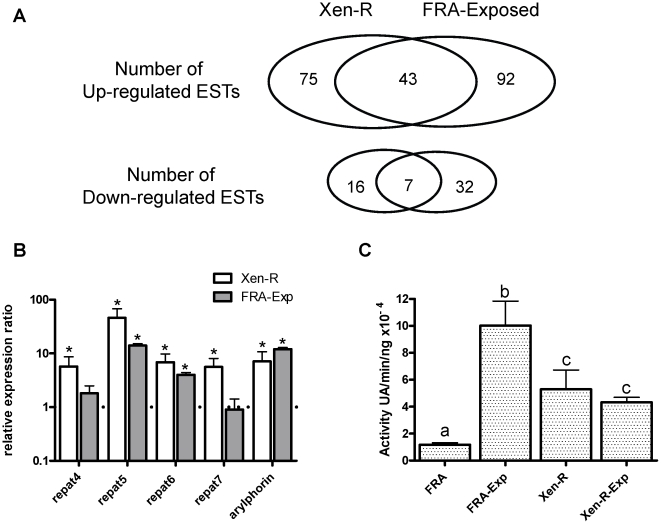
Overlapping characteristics between unexposed Xen-R and exposed FRA insects. A) Schematic representation of the distribution of ESTs differentially expressed in the Xen-R insects and ESTs that are regulated after exposure of FRA insects to Xentari™. B) Relative expression, measured by qRT-PCR, for the selected genes in the Xen-R unexposed insects and for the FRA insects exposed to Xentari™. Asterisks denote changes in expression that are significantly different from the expression in the unexposed FRA insects (*t-test*, *p-value <0.05*). C) Aminopeptidase activity found in the midgut lumen of the Xen-R and FRA insects with and without exposure to *B. thuringiensis* Cry1Ca toxin. Different letter denote statistically different values (*t-test*, *p-value <0.05*).

Our results show the biggest differences between FRA and Xen-R (in the absence of Xentari™ challenge) in expression of pathogen-related genes. We determined how many of the preferentially expressed genes in Xen-R were involved in response to Xentari™. We used the same DNA-macroarray to determine which ESTs were regulated in response to Xentari intoxication in FRA and compared them with ESTs differentially expressed in Xen-R unexposed to Xentari™. Insects from FRA were fed Xentari™ (FRA-Exp) at a concentration that resulted in ca. 99% growth inhibition. Macroarray analysis of the midgut expression pattern (compared with non-exposed insects) revealed the up-regulation of 92 ESTs in response to Xentari™. From these, ca. 47% (43 ESTs) were coincident with ESTs differentially expressed in Xen-R (unexposed) (Fig. 2A and Table S1). Effects of Xentari™ intoxication on gene expression were also measured by qRT-PCR. *Repat5*, *repat6*, and *arylphorin* were up-regulated by intoxication with Xentari™ in FRA, with the highest levels for *repat5* (14-fold) and *arylphorin* (12-fold). In contrast, no significant up-regulation was found for *repat4* and *repat7*. Results are shown and compared with those found for Xen-R (unexposed) in Fig. 2B, and show good agreement between both experiments for *arylphorin*, *repat5* and *repat6*. The overlap observed between ESTs differentially expressed in Xen-R and FRA-Exp suggest that Xen-R has constitutively activated the response to Xentari™.

Recently, Valaitis et al., [28] reported that *B. thuringiensis* toxins trigger the shedding of GPI-anchored aminopeptidase N (APN) from gypsy moth, *Lymantria dispar* midgut epithelial cells. High levels of APN activity in the midgut lumen of Xen-R (unexposed) would also support the hypothesis that midgut response is constitutively activated. To confirm that APN shedding after toxin exposure also occurs in *S. exigua*, we first looked for increases in APN activity in the lumen of FRA exposed to Cry1Ca toxin, the primary *S. exigua*-active toxin present in Xentari. As shown in Fig. 2C, FRA showed a ca. 10-fold increase in APN activity after exposure to a sub-lethal concentration of Cry1Ca toxin. Then we measured APN activity levels in Xen-R with and without exposure to Cry1Ca toxin (Fig. 2C). In agreement with the constitutive midgut activation hypothesis, non-exposed Xen-R showed higher levels of APN activity (ca. 5-fold) compared to non-exposed FRA. However, exposure to Cry1Ca did not have an additional effect on APN levels in the midgut lumen of Xen-R.

### Repat5 and arylphorin overexpression correlates with resistance to Xentari™

Reversion of resistance observed in Xen-RU indicated that resistance was not fixed in Xen-R. Therefore, we expected to find a mixture of susceptible and resistant phenotypes in Xen-RU. If confirmed, this variation could be used to check possible linkages between Xentari™ resistance and gene expression.

**Figure 3:**
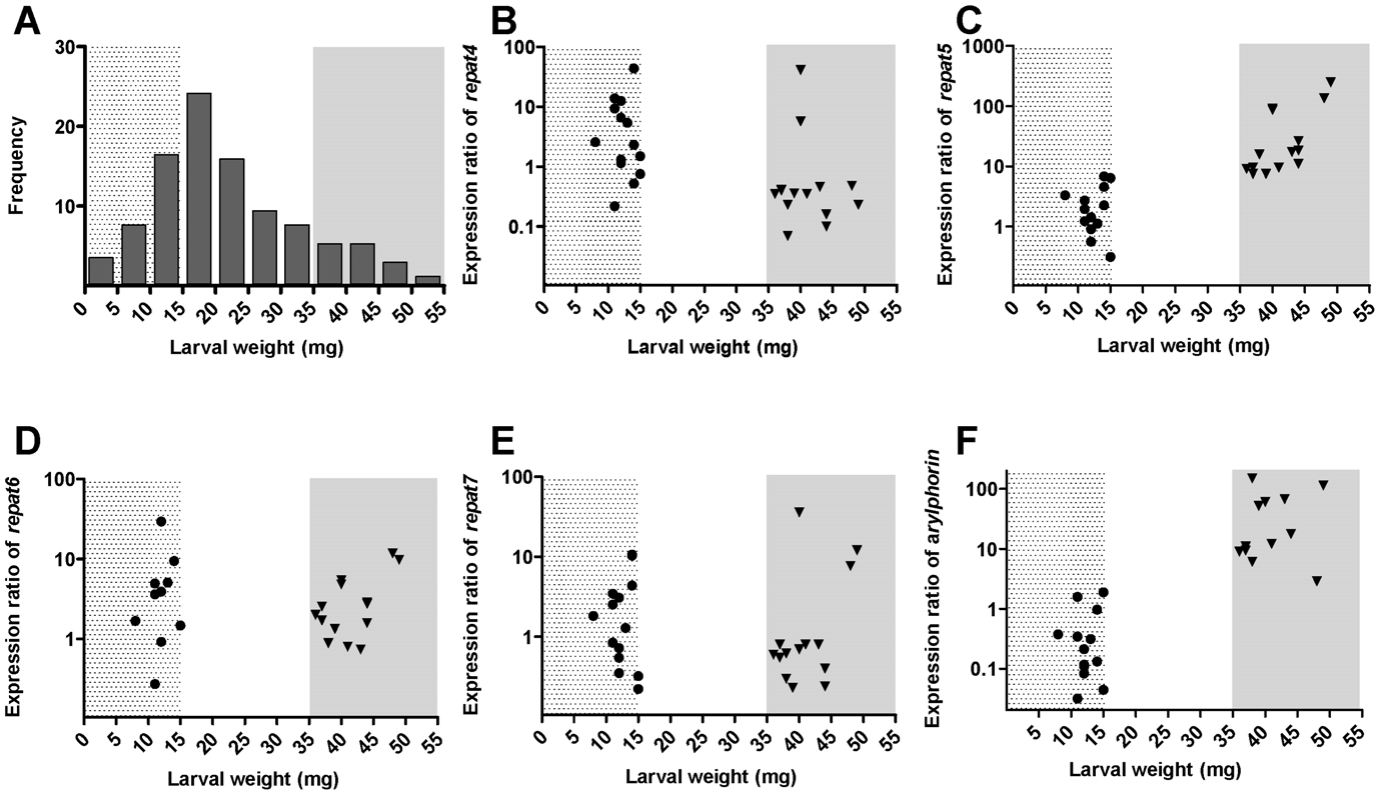
Correlation of resistance and gene expression in the Xen-RU insects. (A) Larval weight distribution (5 mg-intervals) after feeding for 6 days of second-instar larvae on artificial diet containing 25 µg/cm^2^ of Xentari™ product. Two groups of insects were used in further analyses. The S-group (dotted background) included small larvae (susceptible) with a larval weight below 15 mg; the B-group (grey background) included big larva (resistant) with a larval weight above 35 mg. The relative expression, as determined by qRT-PCR, of the genes studied in the two groups of selected larvae is shown in panels B (*repat4*), C (*repat5*), D (*repat6*), E (*repat7*), and F (*arylphorin*). The expression ratio was calculated in relation to the FRA colony using different pools of larvae of similar larval instar and weight for the analysis of the relative expression in the S- or B-group. Results from the correlation analysis between the different pairs are summarized in table 3.

**Figure 4:**
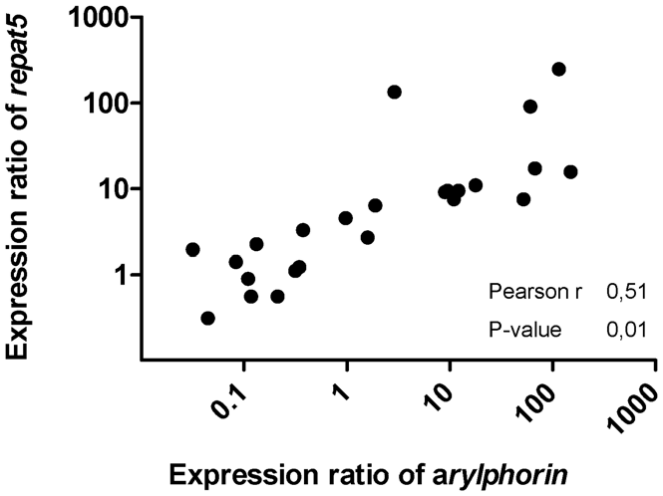
Correlation analysis of *repat5* and *arylphorin* genes. Correlation analysis between the expression ratio of *repat5* and *arylphorin* in individual larvae from the Xent-RU colony (Pearson r and *p-value* are shown).

In order to discriminate between susceptible and resistant individuals, second-instar Xen-RU larvae were fed for 6 d on artificial diet containing a sublethal concentration of Xentari™. Distribution analysis of larval wt (Fig. 3A) revealed a wide growth response in the presence of Xentari™ with values ranging from 4 mg to 55 mg per larva with an average wt of 15.96 (SD = 8.90) mg per larva. Average wt for non-exposed insects was 31.5 (SD = 14.51) mg per larvae, confirming the presence of susceptible as well as resistant phenotypes. Two groups of insects were selected for further analyses. The highly susceptible S-group had larval wts below 15 mg. The highly resistant B-group had larval wts above 35 mg. Insects from both groups were dissected and the relative midgut gene expression was determined using qRT-PCR (Fig. 3B–F and Table 3). Significantly (P<0.05) higher *repat5* and *arylphorin* expression were found from the B-group compared to the S-group, although no differences were detected for *repat4*, *repat6* and *repat7* (Fig. 3 and Table 3). Expression of *repat5* correlated with *arylphorin* expression (Fig. 4), suggesting that both genes encode proteins involved in the same process or controlled by the same elements.

**Table 3:** Gene expression ratio average for the studied genes and correlation analysis between gene expression and larval growth summary.

	Average expression (SD)		Pearson r (*p-value*)^1^
Gene	S-group	B-group	S-group *vs* B-group
*repat4*	7.3 (11.4)	3.6 (10.9)	−0.177 (0.366)
*repat5*	2.4 (2.1)	49.5 (69.9)	**0.546 (0.003)**
*repat6*	5.4 (7.9)	3.5 (3.4)	−0.106 (0.599)
*repat7*	2.9 (3.5)	4.4 (9.7)	0.149 (0.450)
*arylphorin*	0.5 (0.6)	42.7 (47.9)	**0.572 (0.003)**

1 Values in bold denote statistically significant correlation (*p-value <0.05*).

### Midgut proliferation rate is not increased in resistant or susceptible exposed insects

**Figure 5:**
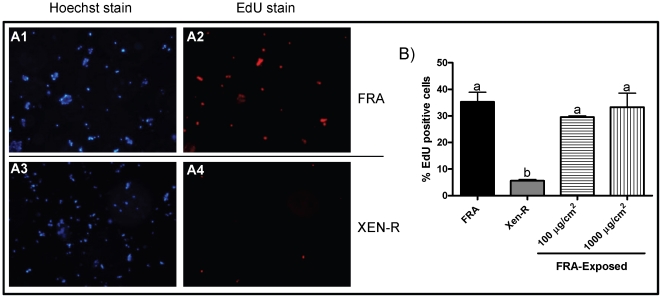
Midgut proliferation in FRA and Xen-R larvae. Hoechst (nuclear DNA) and EdU (DNA synthesis) staining of dissociated cells from last instar larvae midguts from the FRA (A1 and A2) and Xen-R (A3 and A4) colonies. Larvae were injected with EdU reagent and 5 hours post injection, midguts were dissected, cells dissociated and stained for Hoechst and EdU. Percentage of EdU positive cells in FRA, Xen-R, and FRA insects previously exposed to Xentari™ (B). Different letter denote statistically different values (*t-test*, *p-value <0.05*).

Monomeric α-arylphorin has been reported as a mitogenic agent in isolated midgut stem cells from other Lepidoptera such as *Manduca sexta*, *Heliothis virescens*, and *Spodoptera littoralis* [29]; [30] and from the coleopteran *Leptinotarsa decemlineata* [30]. Moreover, recent studies with *D. melanogaster* adults show an increase in midgut epithelial renewal after ingestion of *Erwinia carotovora* or *Serratia marcescens* [20]; [25]. Therefore, we compared midgut proliferation between Xen-R and FRA. Last instar larvae were intrahemocelically injected with EdU (a nucleoside analog) that is incorporated into *de novo*-synthesized DNA. Five hours post injection, midguts were dissected and their cells dissociated and EdU-stained in order to determine levels of DNA synthesis in epithelial cells as a measure of the proliferation rate of intestinal cells [25]; [31]. After 5 h, ca. 35% and 8% of the cells were EdU positive in FRA, and Xen-R, respectively (Fig. 5), indicative of a lower proliferation rate in Xen-R. In order to determine if this reduction in proliferation of intestinal cells could be partly responsible for Xen-R resistance, the effect of Xentari™ exposure on the proliferation rate in FRA larvae was also assessed. No changes in midgut proliferation rate were observed after 7 h of Xentari™ exposure (Fig. 4). No differences were observed (compared with non-exposed insects) for either of the two concentrations tested indicating that FRA larvae do not respond to Xentari™ exposure by activation of midgut epithelium renewal through increased cell proliferation.

## Discussion

With the goal to find genes contributing to the resistance to *B. thuringiensis* containing multiple *S. exigua*-active compounds, we measured differential gene expression between a susceptible *S. exigua* colony and a colony that had developed high levels of resistance to a *B. thuringiensis-*based formulated product (Xentari™). Although neonate selection was discontinued after five days, resistance ratios obtained using both neonates and late instar larvae show that resistance was maintained during the entire larval stage, including the larval instar used in the macroarray analyses.

One of the best-known resistance mechanisms to *B. thuringiensis* is the reduction in binding of *B. thuringiensis* toxins to their specific midgut receptors. Cadherin-like proteins, midgut aminopeptidases and alkaline phosphatases are known as the Cry toxin receptors, and mutations in these proteins are associated with *B. thuringiensis* resistance in several insect populations [32]–[34]. Although the DNA-macroarray used in the present study contained ESTs representing four midgut aminopeptidases [9] and the cadherin-like protein from *S*. *exigua*, no changes in the expression of any of these genes were detected in the Xen-R. These results are indicative of a mechanism other than binding alteration to Cry1C, the primary *S. exigua*-active Cry protein in Xentari™. Nevertheless, the possibility that genes encoding these receptors could carry mutations affecting toxin affinity without affecting their expression cannot be discarded with our analyses.

Among genes differentially expressed in Xen-R, the most significant ones were validated by qRT-PCR, including four *repat* genes (three of them previously undescribed) and one *arylphorin* gene. Up-regulation of *repat1* to *4* was first identified when *S. exigua* susceptible midgut response to Cry1Ca and Cry1A toxin intoxication and infection with baculovirus was analyzed by DNA-microarray (using the same EST library) [9]. Interestingly, although ESTs corresponding to *repat5 -repat7* were also present in the microarray used in the previous study, these genes were not up-regulated in response to Cry1Ca. This different pattern in the expression of *repat* genes found between the exposure to Cry1Ca and the exposure to Xentari™ seems to be indicative of a complex system that determines the type of repat protein that would participate in the response. Apparently, different pathogens or toxic agents would induce the activation of different mechanisms of response involving the action of different repat members. Recently we have obtained ca. 20,000 EST sequences from *S. exigua* larvae exposed to various pathogens using 454-based pyrosequencing. Among these ESTs we found more than 20 members of the *repat* family. This relatively high number of members would be in agreement with the hypothesis that different sets of *repat* genes are activated depending on the type of pathogen or toxin product used. Additional studies on the expression pattern of the different *repat* genes in response to different pathogens/agents would contribute to clarify the reasons for the heterogeneous response that we have observed. Homologs to *repat* in *Spodoptera frugiperda* have also been detected in EST libraries obtained from the insect midgut intoxicated with Cry1C [35] or exposed to xenobiotics [36]. Up-regulation of *arylphorin* has also been detected in the midgut of another Lepidoptera, *T. ni*, after feeding on non-pathogenic bacteria [23].

High levels of expression of *repat* and *arylphorin* genes (pathogen induced genes) suggest that Xen-R could have the response to Xentari™ constitutively-activated even in the absence of infection/intoxication. This hypothesis was additionally supported because more than 50% of the ESTs differentially expressed in Xen-R are also regulated in susceptible insects in response to Xentari™ (Fig. 2 and Table S1). In agreement with this hypothesis we also found higher APN activity in the midgut lumen of the Xen-R insects in the absence of exposure to Xentari™ (Fig. 2). Valaitis et al., [28] proposed that shed APN may promote the cytocidal activity of *B. thuringiensis* rather than being a potential defense mechanism. However, our results with the Xen-R insects are more in favor of the second option. Soluble APNs could act as competitive inhibitors, preventing the interaction of the toxins found in the Xentari™ with the cell surface receptors and contributing to the resistance in Xen-R.

The relevance of the over-expression of *repat5* and *arylphorin* found in Xen-R is supported by the correlation found between gene expression and larval susceptibility to Xentari™ in Xen-RU. Surprisingly, this correlation was not found for *repat4*, *repat6*, and *repat7* suggesting that the contribution of these genes to Xentari™ resistance in Xen-R is not as important as the contribution of *repat5* and *arylphorin*, or their effect might be masked by the effect of the latter genes in our analysis. Expression levels of *repat5* and *arylphorin* also showed a strong correlation, suggesting that both genes may be part of the same biological process.

Arylphorin obtained from the fat body of *M. sexta* larvae showed mitogenic effects on dissociated larval midgut cells and when larvae were fed artificial diet supplemented with arylphorin [29]; [30]. Also, Loeb et al., [37] reported an increase of ca. 2-fold in the percentage of differentiating cells in midgut cell cultures from *H. virescens* after treatment with Cry1Ac toxin. However, recent studies on *D. melanogaster* adults have shown the involvement of midgut epithelium renewal on insect response to infectious bacteria [20]; [24]; [25] or protozoan ookinetes [26]. Based on these observations, exposure of larvae to Xentari™ might induce midgut cell proliferation in *S. exigua* and that Xen-R would have a higher level of midgut cell proliferation when compared to FRA. In contrast to our expectation, Xen-R had a lower midgut cell proliferation rate than FRA. According to the overlap between Xen-R and FRA-exposed larvae, if a reduction in midgut cell proliferation in Xen-R was due to the constitutive activation of midgut response to Xentari™, we should find a decrease in midgut proliferation in FRA-exposed larvae as well. However, we observed that exposure of FRA to different concentrations of Xentari™ had no effect on the midgut proliferation rate. These results suggest that the reduction in midgut proliferation observed in Xen-R may not be related with their resistance and might be a consequence of apparent fitness costs; compared with FRA, Xen-R (unexposed) had a significantly (p<0.05) longer developmental time (15% longer) and smaller pupal size (36% smaller). These results indicate that midgut responses to pathogens may depend on the insect species, larval instar and the pathogen mode of action. Insect larvae spend most of their time eating and experience a continuous renewal of midgut epithelium during each molting step. For instance, ca. 75% of the midgut in *M*. *sexta* is renewed in each molting step [38]. In contrast, adult insect guts are much less dynamic organs and the presence of stem cells has been only recently documented [39]. It is logical to think that different mechanisms of response should operate in these two insect stages. Moreover, recent studies on *D. melanogaster* have shown that midgut response also depends on the type of bacteria. Buchon et al., [24] found that infection with *E. carotovora* does not produce lethal infections in flies but induces an increase in gut renewal. In contrast, gut renewal after ingesting the *Pseudomona entomophila* was dose dependent: lethal bacterial concentrations did not induce an increased rate of gut renewal, but sublethal concentrations did.

Although delta-endotoxins (Cry toxins) are known as the primary virulence factor from *B. thuringiensis*, this bacterium has also developed an arsenal of additional virulence factors such as beta-exotoxins, vegetative insecticidal proteins (Vip), chitinases, zwittermicin, spores and other uncharacterized spore-associated factors [2]; [40]–[43]. All of the above mentioned compounds have been shown to be toxic to *S. exigua*, and at least bacterial spores, Cry toxins, and zwittermicin are found in Xentari™. Thus, in contrast to resistance to single Cry toxins that could be accomplished by modification of a receptor or by changes in the expression of one proteinase, selection for resistance to Xentari™ may require changes in multiple processes that would contribute to overcome all relevant insecticides found in Xentari™. The mechanism(s) of resistance found in Xen-R could be of relative importance for current and future generations of pyramided *B. thuringiensis* crops where multiple insecticidal components with various modes of action are expressed in the same plant [44]. Furthermore, several studies have demonstrated the contribution of plant secondary metabolites (mainly terpenoids) [45]; [46] and proteinases inhibitors [47] to the overall toxicity of plants expressing Cry proteins, or treated with *B. thuringiensis* formulated products suggesting that only mechanism(s) of resistance that could overcome the effects of multiple compounds (as in Xen-R) would be selected for. For example, Cry1Ac-resistant *Helicoverpa zea* was unable to survive on Bollgard cotton, even though it could survive on artificial diet containing Cry1Ac toxin concentrations similar to that expressed in Bollgard cotton; there was a synergistic interaction between the cotton compound gossypol, and Cry1Ac toxin [45]. Whether the activation of response(s) in Xen-R could be attributed to a change in a key gene that activates pathway(s) involved in the response, or the independent activation of different response processes would need to be elucidated. Recently, Cancino-Rodezno et al., [48] have reported in *M. sexta* larvae the activation of the MAPK p38, a master protein that regulates the activity of multiple transcription factors [49], after ingestion of a suspension of *B. thuringiensis* spores and crystals containing Cry1Ab, suggesting that the MAPK p38 pathway is involved in insect defense against *B. thuringiensis*. It would be interesting to determine in further studies if the expression of *repat* genes, *arylphorin* or other differentially-expressed ESTs in Xen-R are under the control of the MAPK p38 pathway.

In summary, we have found a significant overlap between genes that are differentially expressed in Xen-R and genes that are up-regulated after exposure of susceptible insects to Xentari™. Results also show possible associations between REPAT and arylphorin expression (or the genes governing their expression) and resistance to Xentari™, and demonstrate that exposure of larvae to Xentari™ does not have an effect on the midgut epithelium renewal. Additional studies would contribute to identifying the mutation(s) in genes involved in the midgut response to pathogens and to clarify the role of REPAT and arylphorin in response to pathogens.

## Materials and Methods

### Insects rearing, selection and bioassays

*S. exigua* colonies were reared on artificial diet at 25±3°C with 70±5% RH and a photoperiod of 16/8 h (light/dark). Resistant colonies were initiated from ca. 20,000 individuals (100 egg masses) collected from in June-July, 1994 in cotton fields in Prattville, AL, USA. The initial colony was selected for several years with increasing concentrations of Xentari™ containing *B. thuringiensis* subsp. aizawai (Valent Biosciences; containing Cry1Aa, Cry1Ab, Cry1C, Cry1D, and Cry2Ab) in the Department of Entomology, Auburn University, Auburn, AL [50]. Once the resistant colony was established it was maintained with a constant selection protocol. Briefly, neonate larvae were reared for five days on artificial diet containing Xentari™ (10 mg/gram of diet). Survivors were transferred to untreated diet to complete their life cycle. The resistant colony was also sent to the University of Valencia and divided into two different colonies. One colony (Xen-R) was selected every two generations under the same conditions described above in order to maintain selection pressure. The second colony (Xen-RU), used in the correlation experiments, was reared without exposure to the commercial product for at least 8 generations. The FRA colony was kindly provided by M. López-Ferber, INRA (St Christol les Alés, France). This colony has been maintained for at least 10 years without *B. thuringiensis* exposure and was used as the control. Susceptibility to Xentari™ was measured for neonates and fourth instar larvae. Mortality bioassays were performed with neonates using surface contamination as previously described by Herrero *et al*. [51]. Susceptibility of fourth instar larvae was determined in growth inhibition assays using surface contamination as previously described [52]. All assays were repeated at least 3 times for each colony. Resistant ratios (RR) were obtained as the quotient between the LC_50_, IC_50_, and IC_95_ of the resistant colony and its counterpart value for the susceptible colony, respectively.

### DNA-macroarray

The DNA-macroarray was obtained by spotting ca. 600 PCR-amplified ESTs, obtained from *S. exigua* suppression subtractive hybridization derived libraries (GenBank: HO001564-785) obtained in previous studies [9], over positively charged nylon membrane (Amersham Hybond N+). Printing was done with a BioGrid apparatus (BioRobotics, UK) using a 384-pinhead printer, consisting of regular 4×4 spots per pin (0.4 mm diameter). Each PCR product was spotted 5 times for each position (20 nl each time) and each EST was represented in 2 positions on the membrane. After printing, membranes were neutralized with 1.5 M NaCl, 0.5 M Tris/HCl (pH 7.2), 1 mM EDTA (pH 8.0) for 1 min and kept on filter paper until completely dry.

DNA-macroarray experiments were carried using 4^th^ instar larvae from Xen-R and FRA (exposed or non-exposed to Xentari™). Larvae employed for the macroarray experiments were reared in the absence of exposure to Xentari™ until 4^th^ instar. Then, larvae were either fed on artificial diet containing a concentration of Xentari™ estimated to produce 100% growth inhibition (1000 µg/cm^2^) or fed with untreated diet for 24 h. Larvae that consumed diet were dissected and midguts pooled and stored at −80°C. Experiments were independently conducted three times.

Total RNA was extracted from *S. exigua* midguts using Trizol (Invitrogen) following the manufacturer's instructions. The quality and quantity of total RNA were determined spectroscopically at 230, 260, and 280 nm. For each sample, about 30 µg of total RNA were retrotranscribed into cDNA by adding 200 units of RT polymerase SuperScript II (Invitrogen), 500 ng of oligo(dT) primer, 1 µl of RNaseOUT (Invitrogen), 6 µl of 5X First Strand Buffer (Invitrogen), 1.5 µl of dNTP mix (16 mM dATP, dTTP, dGTP, and 100 µM dCTP), and 5 µl of [^33^P]dCTp ( mCi/ml) in a final reaction volume of 30 µl. The labeling mixture was incubated for 1 h at 43°C, and the reaction was stopped by adding 1 µl of EDTA 0.5 M. The radiolabeled sample was purified using an S300-HR MicroSpin columm (Amersham Bioscience).

Hybridization of samples on DNA-macroarrays and data capturing was performed following the protocol described by Belli et al.[53]. Reproducibility of replicates was tested by the ArrayStat software (Imaging Research, Inc.). Differences in gene expression between FRA *vs* Xen-R, and FRA *vs* FRA-exposed were obtained by applying a *Z-test* for independent data (a Z-score was obtained for every gene). A *p-value* ≤0.05 and the False Discovery Rate method were used to monitor the overall false positive error rate and to determine the differentially expressed genes. Macroarray data files can be obtained upon request to the authors.

### Quantitative real-time PCR (qRT-PCR)

Total RNA from different midgut samples used in this study was treated with DNase (Invitrogen) prior to being reverse-transcribed into cDNA using an oligo-dT primer with SuperScript™ II Reverse Transcriptase (Invitrogen). The reverse transcriptase product was diluted 1∶10, and 5 µl were used for each reaction. Specific primers used in qRT-PCR were designed using the software program Primer Express (Applied Biosystems) (Table S2).The amplification reaction was performed using the SYBR Green RT-PCR kit (Appplied Biosystems) on a ABI PRISM 7700 Sequence Detection System (Appplied Biosystems). Expression levels were all normalized against expression levels of a house-keeping gene (ATP-synthase subunit C) and the expression ratios were estimated as described previously [27]: (Xen-R/FRA or FRA-Exp/FRA, 2^ΔΔCt^) where ΔΔCt  =  (Ct _gene a, condition1_ − Ct _ATP-synth, condition1_) – (Ct _gene a, FRA_ − Ct _ATP-synth, FRA_).

### Cloning of novel repat genes

For the cloning of novel *repat* genes a *S. exigua* midgut cDNA library was constructed using the CloneMiner™ cDNA library construction kit (Invitrogen). Gene-specific primers (based on the known EST sequences) and primers from the vector (pDONR222, Invitrogen) used for library construction were employed for PCR-amplification of the 5′ and 3′ fragments. Amplified fragments were cloned into pGemT-easy (Promega). Several clones were sequenced for each fragment and assembled using the Seqman program (DNAstar package). Alignment and phylogenetic tree construction with the deduced amino acid sequences of REPAT proteins was performed using the ClustalX program [54]. Phylogenetic reconstruction was obtained by the neighbor-joining method [55] together with bootstrap analysis using 100 replicates. Kimura correction for multiple substitutions was applied [56].

### Analysis of the correlation between gene expression and resistance

For correlation experiments, 2^nd^ instar larvae from Xen-RU were exposed to a sublethal dose of Xentari™ using surface contamination. Briefly, a total of two hundred synchronous larvae were selected and fed for 6 d on artificial diet containing Xentari™ at 25 µg/cm^2^. At the end of the assay, larvae were weighed individually, midguts dissected, and immediately stored at −80°C for further analysis. Subsequently, final weights of tested larvae were distributed in 5 mg intervals. Midguts from individual insects highly affected (<15 mg of weight; called S-group) and from insects hardly affected by Xentari (>35 mg of weight; called B-group) were used for measuring the expression level of the candidate genes. RNA from midguts were isolated and transcript abundance was obtained by qRT-PCR as described above. Expression ratios were calculated in relation to the FRA colony using different pools of FRA larvae with similar mean wt than the S- and B-group, respectively. Correlation between gene expression level and larval wt (a measurement of resistance) was analyzed using the Pearson correlation coefficient using the GraphPad Prism program (GraphPad software Inc., San Diego).

### Aminopeptidase activity in the midgut lumen

Last instar FRA and Xen-R larvae were exposed to Cry1Ca protoxin (2000 ng/cm^2^) in 50 mM carbonate buffer (pH 10.5) for 12 hours using diet surface contamination [51]. Non exposed insects were treated side-by-side with the same buffer. Midguts were isolated and their contents were obtained by manually discarding of the midgut tissues. The contents were then transferred into 50 mM Tris-HCl pH 8 buffer containing 1 mM PMSF (50 µl/midgut). For each measurement, contents from at least 5 midguts were pooled, vortex mixed for 30 s, centrifuged at maximum speed for 5 min at 4°C and the supernatant was used for activity assays. APN activity was determined using 4 mM L-leucyl-*p-*nitroanilide as substrate and normalized according to the total protein concentration determined by Bradford [57]; [58].

### Determination of midgut cell proliferation rate

Midgut proliferation rate was estimated as the percentage of midgut cells testing positive for *de novo* synthesized DNA after 5 h. DNA synthesis in differentiated enterocytes as well as polyploid epithelial cells was detected using 5-ethynyl-2′-deoxyuridine (EdU; Clik-iT™ Edu Imaging Kit, Invitrogen). Synchronized last instar larvae from FRA and Xen-R were injected intrahemocelically with 15 µl 10% Phenol red solution in PBS containing 0.5 mM EdU. Five hours after EdU injection midguts were dissected and cells dissociated by collagenase (type I; Sigma) treatment. Dissociated cells were fixed and stained for DNA synthesis (EdU) and nucleus (Hoechst 33342) following manufacturer's protocol (Invitrogen). Cells were counted in a fluorescence microscope (DMI3000, Leica Microsystems, Wetzlar). In order to determine whether exposure to Xentari™ modulated the cell proliferation rate, last instar larvae from FRA were previously exposed to Xentari™ at two different concentrations (100 and 1000 µg/cm^2^) by surface contamination. After two h exposure larvae were injected with EdU and processed as described above.

## Supporting Information

Table S1 Differentially expressed ESTs in the Xen-R colony when compared to the FRA colony (Xen-R columm) and their relative change in expression in the FRA colony when exposed to the *B. thuringiensis*-based product, Xentari™ (FRA-E columm).
(0.02 MB PDF)

Table S2 Sequence of the primers employed for quantitative RT-PCR.
(0.01 MB PDF)
