# Supplementary material for: Constitutive Activation of the Midgut Response to Bacillus thuringiensis in Bt-Resistant Spodoptera exigua
Source: PLoS One. 2010 Sep 17;5(9):e12795. doi: 10.1371/journal.pone.0012795 (PMC2941469; doi:10.1371/journal.pone.0012795)
Supplement: Table S1 — Differentially expressed ESTs in the Xen-R colony when compared to the FRA colony (Xen-R columm) and their relative change in expression in the FRA colony when exposed to the B. thuringiensis-based product, Xentari™ (FRA-E columm). (0.02 MB PDF) [file pone.0012795.s001.pdf]

Table S1. Differentially expressed ESTs in the Xen-R colony when compared to the FRA colony (Xen-R columm) and their relative change in expression in the FRA colony when exposed to the *B. thuringiensis*-based product, Xentari™ (FRA-E columm)[1]. Only sequenced ESTs are reported.

EST code	GenBank EST accession nº	Description (BLASTX best hit)	E-value	Xen-R		FRA-E	
				Fold-change	*p-value*	Fold-change	*p value*[3]
**up regulated ESTs**							
Sex_SSH_38	HO001678	Repat5	2.00E-34	45.8[2]	1.0E-04	9.0[2]	1.0E-04
Sex_SSH_437	HO001693	Repat6	8.00E-59	6.8[2]	1.0E-04	2.8[2]	1.0E-04
Sex_SSH_44	HO001694	Repat4	2.00 E-40	5.7[2]	9.6E-02	1.8[2]	NS
Sex_SSH_471	HO001704	Repat7	6.00E-32	5.6[2]	8.3E-03	1.9[2]	NS
Sex_SSH_279	HO004497	Arylphorin subunit	2.00E-41	7.1[2]	2.8E-02	11.9[2]	1.0E-04
Sex_SSH_55	HO001729	No hits found	-	9.8	3.3E-07	3.5	4.4E-05
Sex_SSH_47	HO001702	No hits found	-	8.8	1.5E-07	4.5	2.9E-07
Sex_SSH_334	HO001664	Proteasoma alpha 3	7.00E-21	7.1	1.1E-06	0.9	NS
Sex_SSH_411	HO001683	Proteína ribosomal S11	4.00E-11	7.1	1.0E-06	4.2	9.6E-07
Sex_SSH_451	HO001699	Phospate transport protein	4.00E-25	6.8	1.1E-06	5.1	1.6E-08
Sex_SSH_71	HO001750	Juvenile hormone epoxide hydrolase	8.00E-29	6.7	6.4E-07	6.1	2.4E-09
Sex_SSH_372	HO001675	40S ribosomal protein S6	4.00E-59	6.6	1.7E-06	5.7	2.9E-05
Sex_SSH_200	HO001624	Peritrophin membrane protein 1	5.00E-60	6.2	8.4E-03	3.5	2.9E-05
Sex_SSH_229	HO001633	Fatty acid binding protein	1.00E-21	6.1	1.1E-05	4.5	1.8E-07
Sex_SSH_45	HO001698	No hits found	-	6.1	2.1E-06	3.4	7.2E-05
Sex_SSH_557	HO001734	Trypsin precursor Hz8	9.00E-44	5.5	8.8E-04	2.8	1.4E-03
Sex_SSH_272	HO001650	Moderately methionine rich storage protein	2.00E-47	5.1	3.2E-05	1.0	NS
Sex_SSH_554	HO001732	No hits found	-	5.1	8.7E-04	8.7	3.0E-13
Sex_SSH_136	HO001590	Fatty acid-binding protein 2 (FABP 2)	5.00E-15	5.0	1.2E-04	-2.0	NS
Sex_SSH_486	HO001710	2-hydroxyphytanoyl-CoA lyase	6.00E-70	4.9	2.5E-05	8.9	1.3E-14
Sex_SSH_79	HO001754	Serin proteasa 12	2.00E-30	4.9	7.1E-03	4.5	4.4E-08
Sex_SSH_538	HO001723	No hits found	-	4.8	5.7E-04	3.2	6.0E-04
Sex_SSH_484	HO001709	Heat shock protein	3.00E-7	4.8	2.2E-03	1.2	NS
Sex_SSH_207	HO001625	Mitochondrial prohibitin complex protein 2	6.00E-26	4.5	1.7E-04	3.4	9.7E-06
Sex_SSH_241	HO001637	Ribosomal protein L27A	2.00E-22	4.3	5.0E-04	4.5	6.0E-07
Sex_SSH_122	HO001582	No hits found	-	4.3	1.6E-05	3.5	5.0E-06
Sex_SSH_263	HO001647	No hits found	-	4.3	3.3E-04	1.6	NS
Sex_SSH_81	HO001756	Lipase	2.00E-4	4.2	1.5E-03	3.5	9.7E-05
Sex_SSH_121	HO001581	Ribosomal protein L7A	6.00E-16	4.1	1.5E-04	4.2	1.2E-06
Sex_SSH_72	HO001751	Ribosomal protein S27	3.00E-39	4.1	3.2E-04	3.8	1.4E-05
Sex_SSH_41	HO004498	No hits found	-	4.0	1.2E-04	1.1	NS
Sex_SSH_256	HO001644	Chitin binding PM protein	5.00E-64	3.8	8.5E-04	0.6	NS
Sex_SSH_147	HO001599	Cytochrome c oxidase subunit III	2.00E-48	3.8	3.3E-03	1.4	NS
Sex_SSH_137	HO001591	No hits found		3.5	1.8E-04	1.4	NS
Sex_SSH_129	HO001582	Ribosomal protein S23	1.00E-06	3.5	2.1E-03	4.1	6.5E-07
Sex_SSH_75	HO001753	No hits found	-	3.3	7.5E-04	2.3	2.4E-03
Sex_SSH_266	HO004496	Beta-actin	8.00E-25	3.2	7.2E-03	2.6	1.0E-03
Sex_SSH_59	HO001741	No hits found	-	3.1	9.0E-03	2.8	2.6E-03
Sex_SSH_154	HO001605	Polyubiquitin	6.00E-20	3.1	5.3E-03	4.0	3.5E-07
Sex_SSH_53	HO001721	Putative transmembrane 9 superfamily protein	3.00E-46	2.8	4.2E-03	1.7	NS
Sex_SSH_246	HO001639	Short-chain dehydrogenease/reductase like	9.00E-43	2.8	7.4E-03	1.5	NS
Sex_SSH_161	HO001611	No hits found	7.00E-17	2.7	9.5E-03	1.5	NS
Sex_SSH_249	HO001640	No hits found	8.00E-08	2.7	1.0E-02	1.1	NS
Sex_SSH_531	HO001722	No hits found	-	2.7	1.0E-02	1.8	NS
Sex_SSH_83	HO001758	Repat2	8.00E-37	2.5	2.4E-03	1.7	NS
Sex_SSH_54	HO001724	No hits found	-	2.5	9.0E-03	3.8	2.0E-06
Sex_SSH_172	HO004494	Chymotrypsinogen	8.00E-57	-3.0	5.2E-04	-4.5	5.3E-07
Sex_SSH_101	HO001565	Lipase	1.00E-23	-2.8	2.0E-03	-2.7	3.8E-03
Sex_SSH_174	HO004495	Chymotrypsinogen	3.00E-62	-2.3	3.1E-03	-2.9	1.8E-03
Sex_SSH_550	HO001730	No hits found	-	-2.3	3.1E-03	-2.5	NS
Sex_SSH_138	HO001592	Chymotrypsinogen	6.00E-43	-2.3	1.1E-02	-0.7	NS

[1] Blue marked rows dennote those genes that changed their expression ratio in both comparisons (Xen-R and FRA-E). Not sequenced ESTs have been ommited from the table.

[2]The expression ratio given was obtained by RTqPCR method.

[3] NS, the expression ratio for those genes was statistically not significative (*p-value* > 0.05).
