# Supplementary material for: Constitutive Activation of the Midgut Response to Bacillus thuringiensis in Bt-Resistant Spodoptera exigua
Source: PLoS One. 2010 Sep 17;5(9):e12795. doi: 10.1371/journal.pone.0012795 (PMC2941469; doi:10.1371/journal.pone.0012795)
Supplement: Table S2 — Sequence of the primers employed for quantitative RT-PCR. (0.01 MB PDF) [file pone.0012795.s002.pdf]

Table S2. Sequence of the primers employed for quantitative RT-PCR[1]

Gene name		Primer sequence (5'-3')	Product size (bp)
repat5	Foward	AAGTGGAAATGTGACGCTCCTT	73
	Reverse	CGGGAGGTCCATCAAAAGTC	
repat6	Forward	CGGTTGTTTCCCTGGATGAT	70
	Reverse	GGATGAAATGAGAAGTTATAGCGATCA	
repat7	Forward	TTCGTGCCAACGGTTGAAG	70
	Reverse	TTTTGATGTCACTCTCAGCTCGAT	
arylphorin	Forward	CCGAGGTACCCGCAGTTCT	70
	Reverse	AGCTTTGCATTTTTGTGCGATA	

[1] *repat1*, *repat2*, *repat3*, *repat4* and *ATPsyntase Subunit C* genes were amplified using the pair of primers described by Herrero *et al*. (2007).
